# DNA Aptamer Raised against Advanced Glycation End Products Improves Sperm Concentration, Motility, and Viability by Suppressing Receptors for Advanced Glycation End Product-Induced Oxidative Stress and Inflammation in the Testes of Diabetic Mice

**DOI:** 10.3390/ijms25115947

**Published:** 2024-05-29

**Authors:** Yusaku Mori, Michishige Terasaki, Naoya Osaka, Tomoki Fujikawa, Hironori Yashima, Tomomi Saito, Yurie Kataoka, Makoto Ohara, Yuichiro Higashimoto, Takanori Matsui, Sho-ichi Yamagishi

**Affiliations:** 1 Department of Medicine, Division of Diabetes, Metabolism, and Endocrinology, Anti-Glycation Research Section, Showa University Graduate School of Medicine, Shinagawa 142-8555, Tokyo, Japan; 2 Department of Medicine, Division of Diabetes, Metabolism, and Endocrinology, Showa University Graduate School of Medicine, Shinagawa 142-8555, Tokyo, Japan; ttmichi@med.showa-u.ac.jp (M.T.); shoichi@med.showa-u.ac.jp (S.-i.Y.); 3 Department of Chemistry, Kurume University School of Medicine, Kurume 830-0011, Fukuoka, Japan; higashiy@med.kurume-u.ac.jp; 4 Department of Bioscience and Biotechnology, Fukui Prefectural University, Eiheiji 910-1195, Fukui, Japan

**Keywords:** AGEs, DNA aptamer, diabetes, male infertility, sperm abnormality, testis

## Abstract

Type 2 diabetes mellitus (T2DM) is a risk factor for male infertility, but the underlying molecular mechanisms remain unclear. Advanced glycation end products (AGEs) are pathogenic molecules for diabetic vascular complications. Here, we investigated the effects of the DNA aptamer raised against AGEs (AGE-Apt) on testicular and sperm abnormalities in a T2DM mouse model. KK-Ay (DM) and wild-type (non-DM) 4- and 7-week-old male mice were sacrificed to collect the testes and spermatozoa for immunofluorescence, RT-PCR, and histological analyses. DM and non-DM 7-week-old mice were subcutaneously infused with the AGE-Apt or control-aptamer for 6 weeks and were then sacrificed. Plasma glucose, testicular AGEs, and *Rage* gene expression in 4-week-old DM mice and plasma glucose, testicular AGEs, oxidative stress, and pro-inflammatory gene expressions in 7-week-old DM mice were higher than those in age-matched non-DM mice, the latter of which was associated with seminiferous tubular dilation. AGE-Apt did not affect glycemic parameters, but it inhibited seminiferous tubular dilation, reduced the number of testicular macrophages and apoptotic cells, and restored the decrease in sperm concentration, motility, and viability of 13-week-old DM mice. Our findings suggest that AGEs-Apt may improve sperm abnormality by suppressing AGE–RAGE-induced oxidative stress and inflammation in the testes of DM mice.

## 1. Introduction

Epidemiological studies have reported that 8–12% of couples suffer from infertility globally, and 40–50% of cases are attributed to male infertility [1]. Birth rates have been declining in developed countries [1]; thus, male infertility has appeared as a serious social issue in such societies with declining birth rates and aging populations [1]. Varicocele, hypogonadism, seminal tract obstruction, and sexual dysfunction play a causal role in male infertility, but these disorders account for approximately 40% of the causes of male infertility [2,3]. Therefore, clear causes have not been identified for the rest of patients with male infertility, and these individuals are diagnosed with idiopathic male infertility [2,3], for which a therapeutic strategy is not established.

Diabetes mellitus (DM) is a highly prevalent metabolic disorder globally, and type 2 DM (T2DM) is mainly characterized by hyperglycemia with visceral obesity and insulin resistance, making up approximately 90–95% of DM [4]. Chronic hyperglycemia exposure causes neurovascular dysfunction [5], which results in sexual dysfunction, such as erectile dysfunction and retrograde ejaculation [6]. Indeed, DM has been reported to be one of the established risk factors for sexual dysfunction in males [6]. Furthermore, previous clinical studies have shown that sperm quantity and quality are impaired in males with T2DM [7,8,9,10], thus suggesting that T2DM may be a causal factor of male infertility, regardless of sexual dysfunction. However, the underlying molecular mechanisms relating T2DM to sperm abnormality remain largely unclear [11].

Advanced glycation end products (AGEs) are molecules formed through the nonenzymatic glycation of proteins, lipids, and nucleic acids, and this process has progressed under diabetic conditions [12,13,14]. The macromolecule modification by AGEs alters their structure and function in both animal models and humans [13]. Furthermore, AGEs have been shown to evoke oxidative stress generation and inflammatory responses through the interaction with their cell-surface receptor termed the receptor for AGEs (RAGE) [12,13,14]. AGEs are hardly degraded within the body and are slowly excluded from the kidney, thereby progressively accumulating in the tissues of individuals with DM [15]. Several preclinical and clinical studies have revealed that AGEs play a crucial role in diabetic complication development and progression [12,13,14,15]. Oxidative stress and inflammation have been shown not only to impair spermatogenesis but also damage spermatozoa [16,17]; thus, AGEs may probably have a causal role in sperm abnormalities in T2DM. However, the role of the AGE–RAGE axis in sperm abnormalities in T2DM remains unclear, and the AGE–RAGE pathway inhibition as a potential therapeutic target for DM-associated male infertility remains unclear.

Previously, we revealed that glyceraldehyde-derived AGEs, among various types of AGE structures, mimic the deleterious effects of AGE-rich serum purified from patients with diabetic uremia on neuronal and endothelial cells, and the harmful effects of diabetic serum were neutralized by anti-serum raised against glyceraldehyde-derived AGEs [18,19,20,21]. Furthermore, this type of AGE demonstrated a stronger binding affinity to RAGE, and their levels increased under insulin-resistant and oxidative-stress conditions and were associated with visceral adipose tissue and vascular inflammation in humans [18,19,20,21]. Moreover, seminal fluid contains high levels of fructose, which is metabolized to glyceraldehyde-3-phosphate, thus producing glyceraldehyde-derived AGEs [22,23,24].

Aptamers are composed of short single-stranded DNA or RNA sequences that bind to target molecules and neutralize their functions [25]. Protein antibodies are widely used for the same purpose, but aptamers have many advantages, such as a short generation time, low production costs, less variability between products, and high thermal stability compared with neutralizing antibodies [25]. Currently, many clinical trials are ongoing to assess the efficacy of aptamers against ocular diseases, hematologic diseases, and cancer [25]. Recently, we developed DNA aptamers that have high binding affinity specific to glyceraldehyde-derived AGEs. Furthermore, we revealed that DNA aptamers raised against glyceraldehyde-derived AGEs (AGE-Apts) inhibit the interaction of AGEs with RAGE in vitro [26,27] and hinder the development of diabetic nephropathy and retinopathy, tumor growth, neointimal hyperplasia after balloon angioplasty, and fructose-induced adipocyte remodeling in rodent models [28,29,30,31]. Therefore, the present study investigated the effects of AGE-Apt on testicular and sperm abnormalities in a mouse model of T2DM with obesity and insulin resistance to elucidate the role of the AGE–RAGE axis in male infertility.

## 2. Results

### 2.1. AGE-Apt Inhibited Oxidative Stress, Rage Gene Expression, Inflammation, and Apoptotic Cell Death in the Testes of Diabetic Mice

**Figure 1:**
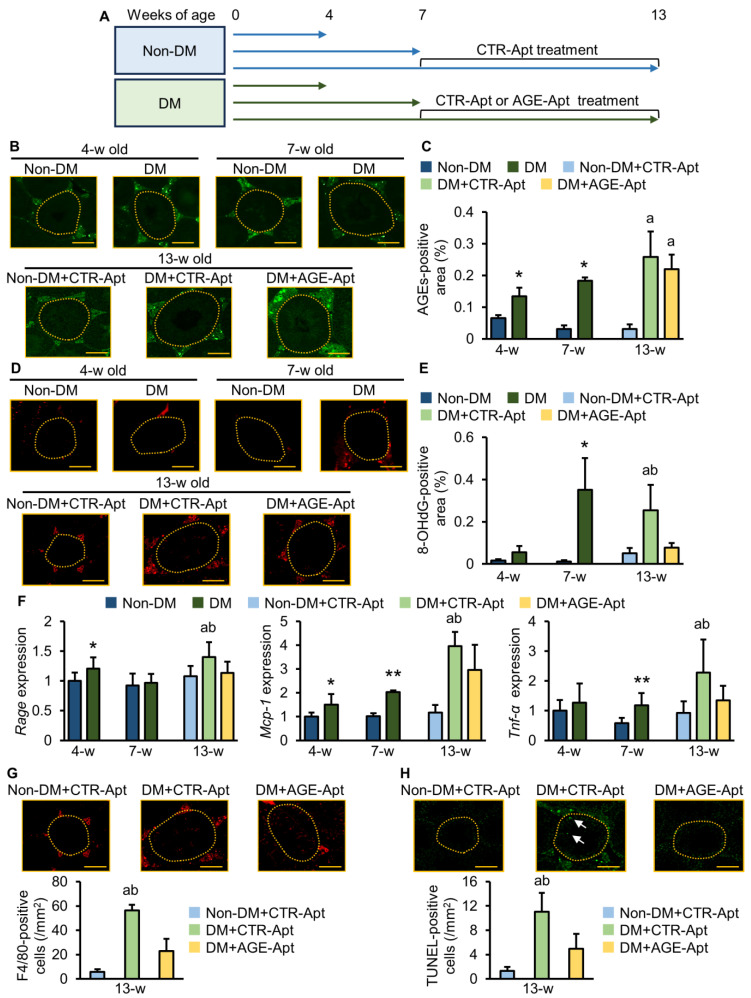
AGE-Apt inhibited oxidative stress and inflammation in the testes of diabetic mice. (**A**) Schema of animal experiments. (**B**) Representative immunofluorescence images for AGEs in each group. (**C**) Testicular AGE accumulation levels. (**D**) Representative immunofluorescence images for 8-OHdG in each group. (**E**) Testicular 8-OHdG levels. (**F**) Testicular gene expression levels of *Rage*, *Mcp-1*, and *Tnf-α* in non-diabetic and diabetic mice. Data exhibit relative levels of target molecules for the housekeeping gene, 18S ribosomal RNA. (**G**,**H**) Number of macrophages (**G**) and apoptotic cells (**H**) in the testes of non-diabetic and diabetic mice. The upper panels indicate representative immunofluorescence images for F4/80 (**G**) and TUNEL (**H**) in each group. (**B**,**D**,**G**,**H**) Yellow dotted lines denote the outer edge of seminiferous tubules, whereas arrows show TUNEL-positive cells within seminiferous tubules. Magnification: ×200; bars: 200 μm. (**C**,**E**,**F**), *n* = 6–8 per group; (**G**,**H**), *n* = 3 per group. * *p* < 0.05, ** *p* < 0.01 vs. non-DM at the same age; ^a^ *p* < 0.05 vs. non-DM+CTR-Apt; ^b^ *p* < 0.05 vs. DM+AGE-Apt. Non-DM: non-diabetic mouse; DM: diabetic mouse; AGEs: advanced glycation end products; CTR-Apt: control aptamer; AGE-Apt: AGE-inhibitory aptamer; w: week; RAGE: receptor for AGEs; MCP-1: monocyte chemoattractant protein-1; TNF-α: tumor necrosis factor-α; TUNEL: terminal deoxynucleotidyl transferase dUTP nick-end labeling.

The experimental schema is shown in Figure 1A. The male mice of non-diabetic C57BL/6J (non-DM) and diabetic KK.Cg-A^y^/TaJcl (DM) mice strains were evaluated at 4, 7, and 13 weeks of age. Body weights and plasma glucose levels were significantly higher, and the testis index was significantly lower in DM mice than in non-DM mice at 4 weeks of age (Table 1). At this age, AGEs already accumulated in the testes of DM mice, which was accompanied by *Rage* and *monocyte chemoattractant protein 1* (*Mcp-1)* gene expression upregulation (Figure 1B,C,F). However, testicular 8-hydroxy-2′-deoxyguanosine (8-OHdG), which is an oxidative stress marker, or *tumor necrosis factor-alpha* (*Tnf-α)* gene levels were comparable between non-DM and DM mice (Figure 1D–F). Glycated hemoglobin (HbA1c) levels, body weights, and plasma glucose levels were significantly higher, and the testis index was significantly lower in DM mice than in non-DM mice at 7 weeks of age (Table 1). Furthermore, oxidative stress and gene expression levels of *Mcp-1* and *Tnf-α* were increased in the testes of DM mice compared with non-DM mice, which were associated with the increased accumulation of AGEs (Figure 1B–F). Food intake, body weight, HbA1c values, and plasma levels of glucose, insulin, total cholesterol, triglycerides, and testosterone were significantly higher, and the testis index was significantly lower in CTR-Apt-treated DM mice than in CTR-Apt-treated non-DM mice at 13 weeks of age (Table 1). Consistent with increased plasma testosterone levels, the testicular gene expression levels of cytochrome P450 family 17 subfamily A polypeptide 1 (*Cyp17a1*), as a marker of the Leydig cell, were increased in CTR-Apt-treated DM mice compared with non-DM mice (Supplementary Materials Figure S1). Furthermore, the number of cells that were positive for F4/80, which is a marker of macrophages, and terminal deoxynucleotidyl transferase dUTP nick-end labeling (TUNEL), which is a marker of apoptotic cells, was increased in the testes of control-aptamer (CTR-Apt)-treated DM mice (Figure 1G,H) accompanied by the increased levels of AGEs, 8-OHdG, *Rage*, *Mcp-1*, and *Tnf-α* gene expressions (Figure 1B–F). AGE accumulation and oxidative stress in the testes of DM mice were only observed in the interstitial area but not within the seminiferous tubules throughout the experimental periods (Figure 1B,D). F4/80-positive cells were also observed only in the interstitial area of the testes in DM mice at 13 weeks of age (Figure 1G), whereas TUNEL-positive cells were found in both interstitial and seminiferous tubular areas (Figure 1H). The 6-week intervention with AGE-Apt did not affect glycemic or metabolic parameters or AGE accumulation levels in DM mice at 13 weeks of age (Table 1, Figure 1B,C), but it significantly reduced 8-OHdG levels, *Rage*, *Mcp-1*, *Tnf-α*, and *Cyp17a1* gene expressions, and the numbers of F4/80- and TUNEL-positive cells in the testes of DM mice (Figure 1D–H and Figure S1).

### 2.2. AGE-Apt Attenuated Seminiferous Tubular Dilation and Sperm Abnormalities in Diabetic Mice

**Figure 2:**
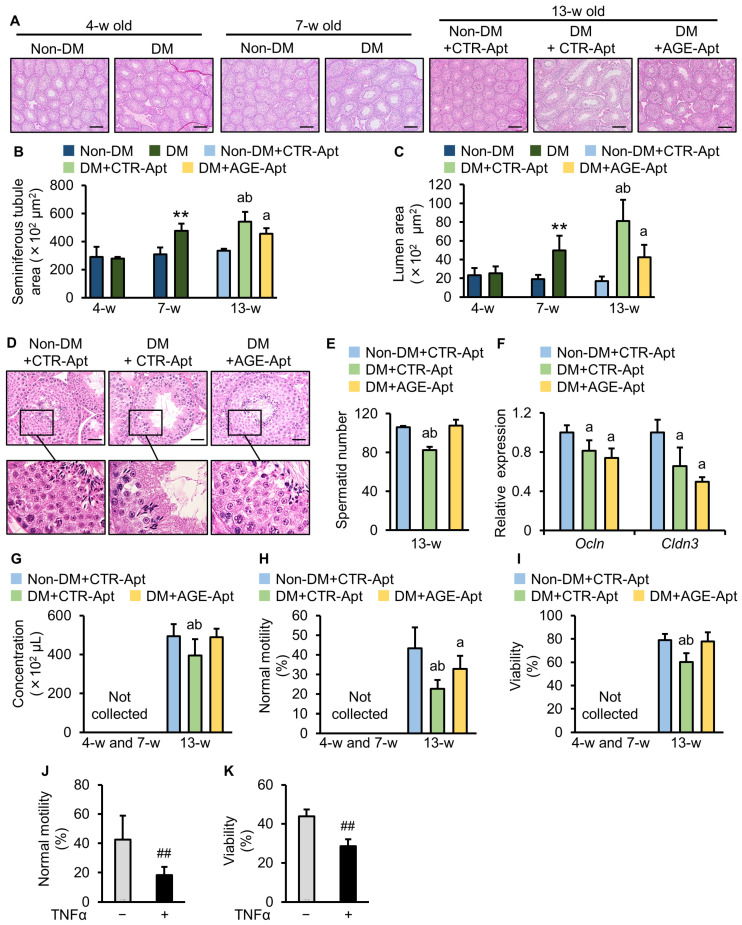
AGE-Apt attenuated seminiferous tubular dilation and sperm abnormalities in diabetic mice. (**A**) Representative images of the seminiferous tubules of non-diabetic and diabetic mice stained with H&E. Magnification: ×40; bars: 200 μm. (**B**,**C**) Seminiferous tubule area (**B**) and lumen area (**C**). (**D**) Representative images of spermatids in the testes of non-diabetic and diabetic mice at 13 weeks of age stained with H&E. Magnification: ×400; bars: 20 μm. (**E**) The number of spermatids. (**F**) Testicular gene expression levels of *Cldn-3* and *Ocln* in non-diabetic and diabetic mice. Data demonstrate relative levels of target molecules to the housekeeping gene 18S ribosomal RNA. (**G**) Sperm concentration. (**H**) Sperm normal motility. (**I**) Sperm viability. (**J**) Sperm normal motility after 6 h of incubation with TNFα. (**K**) Sperm viability after 24 h of incubation with TNFα. (**B**,**C**,**F**–**I**), *n* = 6–8 per group; (**E**), *n* = 4 per group; (**H**), *n* = 6 per group; (**I**), *n* = 3 per group. ** *p* < 0.01 vs. non-DM at the same number of weeks old; ^a^ *p* < 0.05 vs. non-DM+CTR-Apt; ^b^ *p* < 0.05 vs. DM+AGE-Apt; ^##^ *p* < 0.01 vs. control. Non-DM: non-diabetic mouse; DM: diabetic mouse; AGEs: advanced glycation end products; CTR-Apt: control aptamer; AGE-Apt: AGE-inhibitory aptamer; w: week; *Ocln*: *occludin*; *Cldn3*: *claudin-3*.

Histological analysis revealed no significant difference in the seminiferous tubular structure between non-DM and DM mice at 4 weeks old (Figure 2A–C), but significantly dilated seminiferous tubules in the testes of DM mice at 7 and 13 weeks of age were found (Figure 2B) compared to non-DM mice of the same ages, which was associated with an increased luminal area (Figure 2C). Six-week intervention with AGE-Apt significantly attenuated the seminiferous tubular dilation and the increase in the luminal area of the testes of DM mice (Figure 2A–C). At 13 weeks of age, the number of spermatids was significantly decreased in DM mice compared with non-DM mice (Figure 2D,E), which was significantly attenuated by a six-week intervention with AGE-Apt (Figure 2D,E). The assessment of molecules comprising the blood–testis barrier (BTB) revealed decreased gene expression levels of *occludin* (*Ocln*) and *claudin-3* (*Cldn3*) in the testes of CTR-Apt-treated DM mice (Figure 2F). However, AGE-Apt did not affect these gene expressions (Figure 2F).

We then studied the effects of AGE-Apt on sperm parameters. No spermatozoon was collected from non-DM or DM mice at 4 or 7 weeks of age (Figure 2G–I). Sperm concentration, normal motility, and viability were significantly decreased in CTR-Apt-treated DM mice compared with CTR-Apt-treated non-DM mice at 13 weeks of age (Figure 2G–I). The AGE-Apt treatment for 6 weeks significantly restored the decrease in sperm concentration and improved normal motility and the viability of sperm in DM mice at 13 weeks of age (Figure 2G–I).

### 2.3. TNF-α Impaired Sperm Motility and Viability

Finally, spermatozoa collected from non-DM mice at 13 weeks of age were incubated with a vehicle or TNF-α to investigate the direct effects of TNF-α on spermatozoa. Both sperm mobility and viability were significantly decreased by incubation with TNF-α (Figure 2J,K).

## 3. Discussion

Birth rates have been declining in many developed countries, and increasing rates of male infertility have become a crucial social issue in these societies with declining birth rates and aging populations [1]. Clinical studies have revealed that both T1DM and T2DM are associated with sperm abnormalities in males [4]. However, most preclinical studies have investigated the mechanisms underlying DM-induced sperm abnormalities using rodent models of T1DM [11]. T1DM only accounts for <5%–10% of the diabetic population, and most of the diabetic patients are T2DM [4]. T1DM and T2DM feature chronic hyperglycemia, but their pathologies are completely different as follows: the main characteristic of T1DM includes impaired insulin secretion due to insulin-producing pancreatic β-cell destruction, whereas that of T2DM includes insulin resistance caused by visceral obesity [4]. Accumulating evidence has suggested the association of obesity and insulin resistance with male infertility [32]. Furthermore, some T2DM mice models, such as db/db and ob/ob mice, are highly infertile due to the lack of leptin signaling, which is independent of DM [33,34,35], but KK-Ay mice still possess fertility [36,37]. Therefore, to the best of our knowledge, this is the first study to report testicular and sperm changes at sexually pre-mature, mature, and post-mature conditions using a mouse model of T2DM with preserved fertility.

While mice at 7 weeks of age exhibit sexual maturation characterized by testicular and seminal vesicle growth and plasma testosterone levels, the ones at 13 weeks of age have spermatozoa with highly fertilizing potential [38]. In this study, we aimed to compare testicular and sperm changes in sexually pre-matured, matured, and post-matured conditions, which correspond to 4, 7, and 13 weeks of age, respectively. These are the reasons why we evaluated testes and spermatozoa at 4, 7 and 13 weeks of age in the present study. In addition, the overall differentiation process from spermatogonia to mature spermatozoa in mice is reported to be approximately 5 weeks [39]. To investigate the role of AGEs in this process, we administered AGE-Apt to sexually matured 7-week-old mice for six weeks.

**Figure 3:**
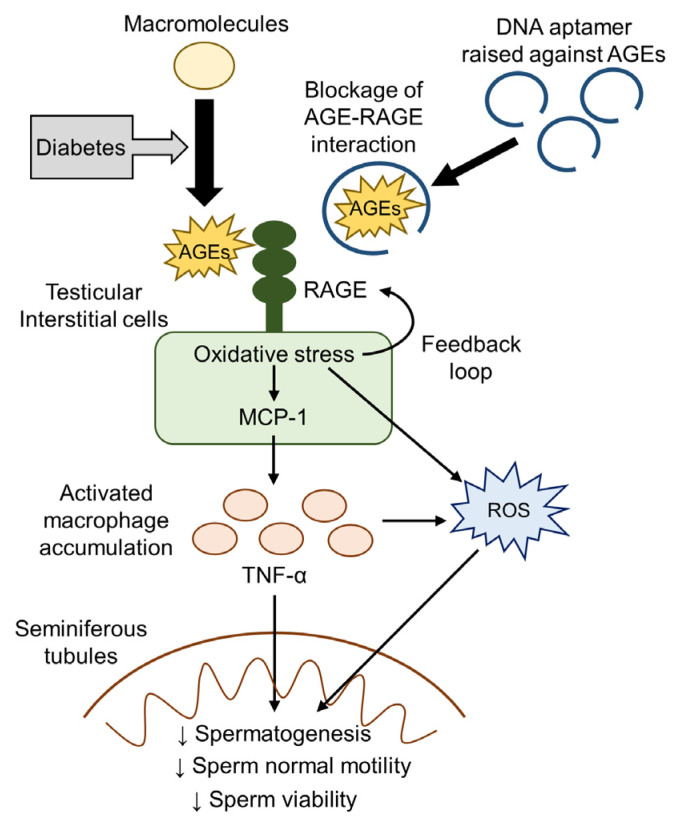
. Proposed mechanism of AGE-induced sperm abnormalities in T2DM and their blockade by AGE-Apt. AGEs: advanced glycation end products; RAGE: receptor for AGEs; MCP-1: monocyte chemoattractant protein-1; TNF-α: tumor necrosis factor-α; ROS: reactive oxygen species.

The present study revealed that body weights and plasma glucose levels were significantly increased in 4-week-old DM mice compared with age-matched non-DM mice, whereas HbA1c levels were comparable between them. HbA1c levels reflect average plasma glucose levels over the preceding 4–8 weeks [4], indicating that DM mice were not exposed to chronic hyperglycemia evaluated by HbA1c at this time point. Conversely, we revealed already increased AGE accumulation levels in the testes of 4-week-old DM mice compared with age-matched non-DM mice. Therefore, AGE accumulation could be stimulated in the diabetic testes under insulin-resistant conditions in our model, part of which was independent of chronic hyperglycemia reflected by HbA1c. Furthermore, 8-OHdG, an oxidative stress marker, and pro-inflammatory *Tnf-α* gene expressions were increased in the testes of 7-week-old DM mice after testicular AGE accumulation, which was accompanied by structural changes in seminiferous tubular dilation. Subsequently, abnormal sperm quantity and quality, such as decreases in sperm concentration, normal mortality, and viability, were observed in 13-week-old DM mice. Notably, the 6-week intervention with AGE-Apt treatment significantly attenuated all of the testicular alterations and sperm abnormalities without affecting glycemic, metabolic, and anthropometric parameters or testicular AGE accumulation levels. These findings indicate that the AGE–RAGE interaction may play a causal role in testicular damage and sperm abnormalities in an animal model of T2DM with insulin resistance and obesity, thus suggesting that AGE-Apt may be a novel therapeutic option for treating male infertility in T2DM (Figure 3).

AGEs have been shown to induce oxidative stress generation and inflammatory responses, such as *Mcp-1* and *Tnf-α* gene expressions, through their interaction with RAGE in various cell types [12,13,14]. Furthermore, AGEs are reported to upregulate RAGE expression via oxidative stress generation, thereby forming a positive feedback loop between AGEs and RAGE-induced oxidative stress [40,41]. The present study revealed increased gene expression levels of *Rage* following AGE accumulation in the testes of 4-week-old DM mice. Testicular *Rage* gene expression once returned to non-diabetic levels in 7-week-old mice, but it was recurrently upregulated in 13-week-old DM mice. Moreover, we revealed that the 6-week intervention with AGE-Apt treatment significantly decreased testicular *Rage* gene expression level, which is concomitant with the suppression of testicular oxidative stress levels and pro-inflammatory gene expressions in 13-week-old DM mice. These observations suggest that increased AGE accumulation levels in the diabetic testes under insulin-resistant and hyperglycemic conditions could stimulate RAGE gene expression via oxidative stress generation in a positive feedback manner, which is a molecular target of the AGE-Apt treatment-induced improvement of seminiferous tubular dilation and sperm abnormalities. This study revealed that testicular AGE accumulation per se was not affected by the 6-week treatment with AGE-Apt, which was consistent with the present finding, indicating that AGE-Apt treatment suppressed oxidative stress and *Rage* upregulation in soleus muscles but did not affect skeletal muscular AGE accumulation in a mouse model of sarcopenia [41]. Accordingly, AGE-Apt functions as a blocker of the binding of AGEs to RAGE, which could subsequently reduce oxidative stress generation and inflammation in the diabetic testes, but it did not sufficiently inhibit oxidative-stress-induced AGE formation under insulin-resistant conditions in our model.

Seminiferous tubules are filled with luminal fluid, with their levels balanced via the production of Sertoli cells and reabsorption by epithelial cells [42,43]. Accordingly, epithelial fluid reabsorption dysfunction causes seminiferous tubular dilation by outward pressure overload through luminal fluid accumulation, which damages germinal cells and ultimately impairs spermatogenesis [42,43]. A previous study has revealed that administration of the phosphodiesterase 4 inhibitor, BYK169171, induces seminiferous tubule dilation, which follows testicular inflammation in rats [44]. The present study revealed that seminiferous tubular dilation following an increased liminal area occurred simultaneously with oxidative stress generation and *Tnf-α* gene overexpression in the testes of 7-week-old DM mice despite the absence of the structural abnormality of seminiferous tubules in the testes of 4-week-old DM mice. Additionally, a decreased testicular spermatid number and sperm concentration was observed in 13-week-old DM mice, which was restored by AGE-Apt in association with the inhibition of oxidative stress generation, testicular *Mcp-1* and *Tnf-α* gene expressions, and seminiferous tubular dilation. Seminiferous tubular dilation may be caused by oxidative stress generation and inflammation rather than hyperglycemia per se in our model because AGE-Apt treatment did not affect glycemic or metabolic parameters. Therefore, the present findings suggest the involvement of testicular oxidative stress generation and inflammation evoked by AGE–RAGE axis activation in seminiferous tubular dilation and sperm abnormalities in T2DM.

MCP-1 is a chemokine that plays an important role in inflammatory process initiation by inducing tissue macrophage recruitment [45]. We previously revealed that AGEs increase MCP-1 protein levels in human cultured endothelial and mesangial cells [46,47]. Furthermore, AGEs activate macrophages to produce pro-inflammatory cytokines, such as TNF-α, via their interaction with RAGE [48,49]. An in vitro study revealed that TNF-α reduced the motility and viability of human spermatozoa in a dose- and time-dependent manner [50,51]. Additionally, germ cell apoptosis is shown to be induced by its incubation with the conditioned media of testicular macrophages, whose effect is inhibited by the neutralization of TNF-α [52]. These observations indicate that TNF-α produced by infiltrated macrophages may disrupt sperm production and function. The present study revealed that *Mcp-1* gene upregulation was first observed in the testes of DM mice, which was followed by *Tnf-α* gene upregulation. AGE-Apt administration not only inhibited macrophage accumulation in the testicular interstitial area but also reduced apoptotic cells within the seminiferous tubules, both of which were concomitant with the suppression of testicular *Mcp-1* and *Tnf-α* gene expression levels. Additionally, TNF-α directly impaired the normal motility and viability of sperm in the ex vivo experiments. Therefore, our present findings suggest that, in KK-Ay mice, AGEs could induce macrophage infiltration into the testicular interstitial areas through MCP-1 upregulation via RAGE-induced oxidative stress, which may subsequently evoke germinal apoptotic cell death and sperm dysfunction in seminiferous tubules via TNF-α production (Figure 3).

Accumulating evidence has shown that oxidative stress is an important factor for the development of male infertility in cooperation with inflammation [16]. Spermatozoa are shown to be highly susceptible to the oxidative stress induced by exposure to reactive oxygen species (ROS) because of very low levels of antioxidative enzymes [16]. Physiological ROS levels are required for the acquisition of sperms’ fertile function [16], whereas excessive ROS levels mainly produced from activated leukocytes and macrophages can cause plasma membrane lipid peroxidation, DNA damage, mitochondrial dysfunction, and apoptosis via sperm oxidative stress induction [53]. Sperm oxidative stress and seminal plasma ROS levels are shown to be correlated with sperm abnormalities, such as decreases in sperm concentration, motility, viability, and normal morphology [54,55,56,57,58,59,60,61]. A previous study has shown that diabetic rats exhibit increased testicular ROS levels and impaired sperm parameters, which are prevented by the combination therapy of antioxidants [62]. Consistently, sperm DNA damage is reported to be increased in males with diabetes compared to those without diabetes [63]. As mentioned above, we noted that oxidative stress levels were increased in the testicular interstitial area of diabetic mice, which were consistent with AGE accumulation and macrophage infiltrations. On the other hand, these changes were not observed within the seminiferous tubules. These findings suggest that testicular interstitial cells and infiltrated macrophages may be the major source of ROS production in our model, which is a molecular target by AGE-Apt (Figure 3).

BTB is a complex cell structure present in the seminiferous epithelium, which works as a physiological barrier and is composed of tight, gap, and adhesion junctions between Sertoli cells [64]. BTB plays an essential role in spermatogenesis and sperm function by controlling substance entry into seminiferous tubules [64]. Previous studies revealed that protein or gene expression levels of BTB-composing molecules were decreased in DM animals, thereby causing BTB dysfunction [65]. In the present study, gene expression levels of *Ocln* and *Cldn3* were decreased in the testes of 13-week-old DM mice; however, AGE-Apt did not affect the expressions. Therefore, BTB-composing molecule reductions may be induced by other factors than AGEs: hyperglycemia and/or dyslipidemia may destroy BTB. This may be one of the reasons why AGE-Apt treatment did not fully restore the decrease in sperm motility.

The present study has several limitations. First, plasma testosterone and testicular *Cyp17a1* gene expression levels were higher rather than lower in our DM mice than in non-DM mice, suggesting Leydig cell hypertrophy. However, this change was in contrast with the finding of decreased plasma testosterone levels in males with T2DM [66,67]. Therefore, the mechanisms underlying Leydig cell hypertrophy in our model and the involvement of the AGE–RAGE axis in sperm abnormalities in T2DM with low plasma testosterone remains unclear. Second, we evaluated sperm abnormalities using spermatozoa directly collected from cauda epididymis. However, post-testicular factors, such as seminal plasma, also affect sperm quality in ejected semen [6,7]. Thus, the effects of the AGE–RAGE axis on ejected semen and its contribution to sperm abnormalities in our model remains unknown. Third, we did not evaluate sperm DNA oxidation due to technical difficulty in measuring it. Therefore, it was unclear whether sperm DNA oxidation was increased in DM mice or if it was prevented by AGE-Apt treatment in our model. Fourth, mice at 4 weeks of age are not sexually mature [38]. After spermatogenesis in the testis, spermatozoa acquire motility and fertilizing capacity by passing the caput and corpus regions of the epididymis, and functionally matured spermatozoa are stored in the caudal region [68]. Therefore, although mice at 7 weeks of age are considered sexually mature [38], mice at 7 weeks of age might still be too young to collect spermatozoa from the cauda epidydimides via the swim-out method. These are the reasons why spermatozoa were not collected from mice at 4 or 7 weeks of age. Fifth, perfusion fixation was not performed prior to testes collection because the right testes and epididymides needed to be collected without fixation for mRNA and sperm extraction. Since perfusion fixation is the best way to fix the testes, the lack of this procedure might have affected the histological evaluation of the testes.

## 4. Materials and Methods

### 4.1. Preparation of DNA Aptamers

DNA aptamers were synthesized as previously described [30]. The sequences of DNA aptamers were as follows: AGE-Apt, 5′-tgTAgcccgAgTATcATTcTccATcgcccccAgATAcAAg-3′; CTR-Apt, 5′-aTcgAccTggAggcgAgcAgcTcggATccAg-TcgcgTgAg-3′. Phosphorothioate nucleotides are indicated in capital letters.

### 4.2. Animal Study

The Animal Care Committee of Showa University School of Medicine (approval number: 03034; approval date: 1 April 2021) approved the animal experiments, with adhesion to ARRIVE 2.0 guidelines and the Guide for the Care and Use of Laboratory Animals (8th Edition) [69,70]. Invasive procedures were conducted under general anesthesia using isoflurane. CLEA Japan (Meguro, Tokyo, Japan) supplied 30 male mice of the KK-Ay strain mice, a mouse model of T2DM [36,37], and 22 male mice of the wild-type C57BL/6J strain used as non-DM controls. The mice were maintained in an individual cage with free access to standard rodent chow and water in the animal care facility of Showa University.

Figure 1A shows the experimental schema. Some KK-Ay and wild-type mice were sacrificed at 4 and 7 weeks of age. The rest of the KK-Ay mice at 7 weeks of age were randomly categorized into CTR-Apt or AGE-Apt treatment groups following their cage number. The rest of the non-DM mice at 7 weeks of age were assigned to the CTR-Apt treatment group. Aptamers were continuously infused in mice at 10 pmol/g body weight/day for 6 weeks via osmotic pumps implanted under the dorsal skin [41].

Both sides of the epididymides and testes were carefully excised at the end of each experiment. The epididymides were used to collect spermatozoa. The left testis was soaked in a Bouin solution of hematoxylin and eosin (H&E) and immunofluorescence staining after weighing the testes, and the right one was snap-frozen with liquid nitrogen for the reverse transcription polymerase chain reaction (RT-PCR) assay.

### 4.3. Measurement of Biochemical Parameters, Blood Pressure, and Heart Rates

Plasma levels of biochemical parameters, blood pressure, and heart rates were measured as previously described [71]. Blood samples were collected under a fasting state at the end of each experiment. An immunoassay and an enzyme electrode assay were used for HbA1c and plasma glucose level measurements, respectively. Plasma insulin and testosterone levels were determined with the enzyme-linked immunosorbent assay (Ultra-sensitive Mouse insulin ELISA kit, Product ID: M1104, Morinaga Institute of Biological Science, Yokohama, Kanagawa, Japan; Testosterone ELISA kit, Product ID: ENZ-ADI900065, Enzo Life Sciences, Farmingdale, NY, USA). A non-invasive tail-cuff method was used for systolic blood pressure and heart rate measurements.

### 4.4. Immunofluorescence Staining

Immunofluorescence staining was performed as previously described [71]. Cross-sections were incubated with the anti-AGE antibody (raised in rat, 1: 100) [28], anti-8-OHdG antibody (Product ID: MOG-020P, RRID: AB_1106819, raised in mouse, 1:200; Nikken Seil, Fukuroi, Shizuoka, Japan), or anti-F4/80 antibody (Product ID: Ab204467; RRID: AB_2810932, raised in rat; 1:250; Abcam Japan, Chuo, Tokyo, Japan) overnight. The cross-sections were further incubated with secondary antibodies for 4 h and mounted with VECTASHIELD Antifade Mounting Medium (Vector Laboratories, Newark, CA, USA). Cross-sections were used for the TUNEL assay to detect apoptotic cells, following the manufacturer’s instructions (Product ID: 11684795910; Sigma-Aldrich Japan, Meguro, Tokyo, Japan). A confocal microscope (BZ-X710 microscope Keyence, Osaka, Osaka, Japan) and Image J software (version 1.52v) were used to obtain and analyze immunofluorescence images, respectively.

### 4.5. Real-Time RT-PCR

Total RNA extracted from cells and tissues was used to synthesize cDNA for RT-PCR assay, as previously described [71]. The TaqMan gene expression assay and sequence detection system (Quantstudio3; Life Technologies Japan, Minato, Tokyo, Japan) were used for quantitative real-time RT-PCR [71]. The following pre-designed TaqMan probe sets were used for the assay: *Cldn3*, Mm00515499_s1; *Cyp17a1*, Mm00484040_m1; *Mcp-1*, Mm00441242_m1; *Ocln*, Mm00500912_m1; *Tnf-α*, Mm00443258_m1; *Rage*, Mm00545815_m1; 18S ribosomal RNA (*18s rna*), Mm03928990_g1. The expression levels of the target gene were normalized with those of the internal control *18s rna*.

### 4.6. Histological Assessment of Testis

The left testes were fixed in Bouin solution for 24 h at 4 °C [62,68,72] and then rinsed in 10% neutral buffered formalin for 48–72 h at 4 °C to remove excess picric acid of the Bouin solution, which can interfere with immunofluorescence staining via autofluorescence. After rinsing, the testes were mounted in a paraffin block after being soaked in Bouin solution for 24 h at 4 °C. Cross-sections were obtained from the middle part of paraffin-embedded testes and stained with H&E for measuring seminiferous tubule and lumen areas and the seminiferous tubular spermatid number [72,73]. Image J software (version 1.52v; National Institutes of Health, Bethesda, MD, USA) was used for image analysis. Horizontal sections of seminiferous tubules were randomly chosen for the measurement. At least 20 seminiferous tubules per testis were measured for seminiferous tubule and lumen areas. The number of round spermatids exhibiting condensed nuclei was counted in 10 seminiferous tubules per testis [73]. The averaged data were used as an individual value.

### 4.7. Sperm Collection

The swim-out method was used to collect spermatozoa from the cauda epididymis, as previously reported with some modifications [74]. The left and right sides of the cauda epididymis of each mouse were separately soaked in 1 mL of the M2 medium (Sigma-Aldrich Japan, Meguro, Tokyo, Japan) maintained at 37 °C. Subsequently, the cauda epididymides were minced within the medium using spring scissors, incubated to allow spermatozoa to swim out for 15 min, and then removed from the medium. Spermatozoa were further incubated in the medium for 45 min to induce their capacitation.

### 4.8. Sperm Analysis

Sperm parameters were assessed, as previously reported, with some modifications [62,74,75]. Sperm concentration was counted using hemocytometers. Sperm suspension of 10 μL was diluted with the same volume of M2 medium on a glass slide and covered by a 24 mm × 32 mm cover glass for the sperm motility assessment [74,75]. Individual spermatozoons were microscopically categorized into normal (progressive movement) or abnormal (non-progressive or no movement) motility [74,75]. Sperm viability was identified with eosin-nigrosine staining; 5 μL of the sperm suspension was mixed with 5 μL of a 5% eosin solution for 30 s, and then with 10 μL of 10% nigrosine for an additional 30 s on a glass slide, which was subsequently smeared and air-dried [62,75]. A spermatozoon with an eosin-positive head was counted as a dead cell. Sperm motility and viability were evaluated on at least 100 spermatozoa per epididymis. Spermatozoa that were collected from the left and right epididymides of each mouse were separately analyzed and then averaged as an individual value.

### 4.9. Ex Vivo Assay

Spermatozoa were collected from non-DM mice as previously described. Spermatozoa were seeded into tubes at a 4 × 10^6^ cells/mL density using the M2 medium [50,51] and incubated with a vehicle or recombinant human TNF-α (100 ng/mL; R&D Systems, Minneapolis, MN, USA) at room temperature for 6 and 24 h to assess sperm normal motility and viability, respectively.

### 4.10. Statistical Analysis

The sample size was calculated to minimize the number of animals based on our previous studies [28,29,30,31,41]. Data are expressed as the mean ± standard deviation. JMP software (version 13; SAS Institute, Cary, NC, USA) was used for statistical comparison. Comparisons were conducted using an unpaired *t*-test or one-way analysis of variance with Tukey’s post hoc test as appropriate. The significance level was defined as a *p*-value of <0.05.

## 5. Conclusions

Our present findings suggest the involvement of testicular AGE accumulation in seminiferous tubule dilation and sperm abnormality via testicular oxidative stress and inflammation evoked by RAGE activation in the mouse model of T2DM. The inhibition of the AGE–RAGE axis by AGEs-Apt can be a potential therapeutic option for treating male infertility in T2DM.

## Figures and Tables

**Table 1:** Anthropometric and biochemical parameters of non-diabetic (non-DM) and diabetic mice (DM) at 4, 7, and 13 weeks of age.

	4 Weeks Old		7 Weeks Old		13 Weeks Old		
	Non-DM	DM	Non-DM	DM	Non-DM+CTR-Apt	DM+CTR-Apt	DM+AGE-Apt
Number	7	6	7	8	8	8	8
Food intake (g/day)	N.A.	N.A.	N.A.	N.A.	5.0 ± 0.5	7.3 ± 0.6 ^a^	7.1 ± 0.4 ^a^
Body weight (g)	18.4 ± 1.1	22.5 ± 1.6 *	21.4 ± 1.0	35.8 ± 2.6 *	27.0 ± 1.2	44.0 ± 2.2 ^a^	42.4 ± 1.5 ^a^
Testis weight (mg)	132 ± 18	111 ± 8	190 ± 9	206 ± 21	213 ± 7	205 ± 25	218 ± 13
Testis index (Testis weight [mg]/body weight [g])	7.2 ± 0.6	5.0 ± 0.3 *	8.9 ± 0.5	5.8 ± 0.5 *	7.9 ± 0.3	4.7 ± 0.6 ^a^	5.2 ± 0.4 ^a^
HbA1c (%)	<4.0	<4.0	4.7 ± 0.3	7.0 ± 0.9 *	4.8 ± 0.2	10.2 ± 1.0 ^a^	10.4 ± 1.0 ^a^
Plasma glucose (mg/dL)	160 ± 11	201 ± 16 *	142 ± 14	207 ± 38 *	155 ± 9	190 ± 29 ^a^	174 ± 22
Plasma insulin (ng/mL)	N.A.	N.A.	N.A.	N.A.	0.24 ± 0.06	2.44 ± 1.77 ^a^	1.93 ± 1.17 ^a^
Plasma total cholesterol (mg/dL)	N.A.	N.A.	N.A.	N.A.	38 ± 3	68 ± 5 ^a^	62 ± 11 ^a^
Plasma triglycerides (mg/dL)	N.A.	N.A.	N.A.	N.A.	39 ± 9	115 ± 19 ^a^	105 ± 36 ^a^
Plasma testosterone (ng/mL)	N.A.	N.A.	N.A.	N.A.	1.6 ± 1.1	35.6 ± 26.0 ^a^	26.6 ± 11.3 ^a^

Means ± standard deviation. HbA1c: glycated hemoglobin A1c; N.A.: not available. * *p* < 0.05 vs. non-DM at the same age (weeks); ^a^ *p* < 0.05 vs. non-DM+CTR-Apt.

## Data Availability

Data are contained within the article.

## Supplementary Materials

The following supporting information can be downloaded at https://www.mdpi.com/article/10.3390/ijms25115947/s1.
