# Supplementary material for: DNA Aptamer Raised against Advanced Glycation End Products Improves Sperm Concentration, Motility, and Viability by Suppressing Receptors for Advanced Glycation End Product-Induced Oxidative Stress and Inflammation in the Testes of Diabetic Mice
Source: Int J Mol Sci. 2024 May 29;25(11):5947. doi: 10.3390/ijms25115947 (PMC11172898; doi:10.3390/ijms25115947)
Supplement: Supplementary file 1 [file ijms-25-05947-s001.zip › ijms-3009063-supplementary.pdf]

Supplemental Fig. 1

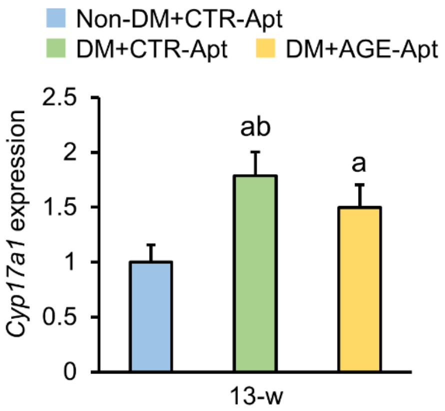

Testicular *Cyp17a1* gene expression levels in non-diabetic and diabetic mice at 13 weeks of age. Data exhibit relative levels of the target molecule to the housekeeping gene, 18S ribosomal RNA. $n = 6–8$ per group. [a]$p < 0.05$ vs. Non-DM+CTR-Apt; [b]$p < 0.05$ vs. DM+ AGE-Apt. Non-DM: non-diabetic mouse; DM: diabetic mouse; CTR-Apt: control aptamer; AGE-Apt: AGE-inhibitory aptamer; w: week; Cyp17a1: cytochrome P450 family 17 subfamily A polypeptide 1.
